# Increasing Vegetable Diversity Consumption Impacts the Sympathetic Nervous System Activity in School-Aged Children

**DOI:** 10.3390/nu13051456

**Published:** 2021-04-25

**Authors:** Francisca de Castro Mendes, Inês Paciência, João Cavaleiro Rufo, Mariana Farraia, Diana Silva, Patrícia Padrão, Luís Delgado, Vanessa Garcia-Larsen, André Moreira, Pedro Moreira

**Affiliations:** 1 Serviço de Imunologia Básica e Clínica, Departamento de Patologia, Faculdade de Medicina da Universidade do Porto, 4200-319 Porto, Portugal; francisca_castromendes@hotmail.com (F.d.C.M.); inespaciencia@gmail.com (I.P.); mariana.farraia@gmail.com (M.F.); disolha@gmail.com (D.S.); ldelgado@med.up.pt (L.D.); andremoreira@med.up.pt (A.M.); 2 EPIUnit—Instituto de Saúde Pública, Universidade do Porto, 4050-091 Porto, Portugal; jcrufo@gmail.com (J.C.R.); patriciapadrao@fcna.up.pt (P.P.); 3 Serviço de Imunoalergologia, Centro Hospitalar São João, 4200-319 Porto, Portugal; 4 Faculdade de Ciências da Nutrição e Alimentação, Universidade do Porto, 4150-180 Porto, Portugal; 5 Program in Human Nutrition, Department of International Health, The Johns Hopkins University Bloomberg School of Public Health, Baltimore, MD 21205, USA; vgla@jhu.edu

**Keywords:** autonomic nervous system, pupillometry, children, dietary diversity

## Abstract

Evidence about the impact of vegetable and fruit diversity consumption on the autonomic nervous system (ANS) functioning is scarce. In this cross-sectional study (513 participants, 49.9% girls aged 7 to 12 years), we evaluated the association between vegetable and fruit diversity consumption and the ANS in school-aged children. Dietary intake was collected using a single 24-h recall questionnaire. Fruit and vegetable diversity consumption was estimated by summing up all the different individual vegetables and fruits consumed in one day. Pupillometry was used to assess pupillary light response, which evaluated the ANS activity. Adjusted linear regressions estimated the association between vegetable and fruit diversity consumption with pupillary light response measures. There was a positive and significant association between vegetable diversity consumption and the average dilation velocity, a measure related to the sympathetic nervous system activity (β-coefficient = 0.03, 95%CI: 0.002; 0.07). Our findings show that vegetable diversity consumption is associated with the ANS response, a possible early link between diet and health in school-aged children.

## 1. Introduction

Over the past decades, there have been changes in children’s dietary patterns [1], which have been characterized by a higher intake of foods with low nutritional value, with increasing processing, and with contents such as fats, added sugars, food additives, and salt. Diets with these Western characteristics are frequently unhealthy and have been associated with an increased risk of several non-communicable diseases [2]. On the other hand, healthy diets, frequently linked with the Mediterranean diet, with higher intake of fruits and vegetables, seem to have a protective effect on cardiovascular health [3] and metabolic diseases [4]. This is particularly relevant considering that these diseases start early in life, where dietary habits are defined [5].

Diet diversity is a key dimension of diet quality [6]. The importance of studying this dimension is related to the fact that foods and nutrients are eaten together and might have more relevance on human biological systems than foods or nutrients alone [7]. In fact, increasing the intake of different food groups might have a synergistic effect, due to possible interactions between them, which might be a potential mechanism to reduce anti-inflammatory and anti-oxidant processes related to several non-communicable diseases [8]. Nonetheless, results from the pre-school children in four European cohorts, including a Portuguese birth-cohort, showed that the majority of the included participants did not achieve the recommended variety of healthy foods consumed [9]. In respect to vegetables and fruits, increasing the consumption of different varieties provides wider sources of different vitamins, minerals, and phytochemicals [10], which are important in anti-oxidant and anti-inflammatory defense processes. Moreover, it has been demonstrated that having a greater diet quality is associated with higher consumption of fruits and vegetables [11]. Our group recently showed that higher vegetable diversity consumption decreased the odds of having higher levels of exhaled nitric oxide and asthma [12]. In addition, another cross-sectional study with Portuguese adolescents found a negative association between a higher variety of vegetables and inflammatory biomarkers, such as C-reactive protein, while a variety of fruit was positively associated with an inflammatory score [13].

The autonomic nervous system (ANS) is a central regulatory system involved in the homeostatic regulation, at short and long term, of several organs and tissues [14]. Considering its role on homeostatic regulation, an autonomic dysfunction has been associated with several diseases, including obesity [15] and cardiovascular disease [16], and these diseases’ pathophysiology includes chronic inflammation. In fact, the ANS seems to influence the anti-inflammatory responses, while it regulates the intestinal barrier function [17]. Moreover, a recent systematic review and meta-analysis also showed that weight gain and loss was associated with a higher activity of sympathetic and parasympathetic nervous system (SNS and PSNS), respectively [18].

Regarding the role of diet on ANS, the majority of evidence comes from experimental studies and suggests that anti-oxidant and polyphenols might reduce the over activity of the sympathetic nervous system, which might be a potential mechanism by which diet protects cardiac health [19]. A twin male study showed a positive association between the adherence to the Mediterranean diet and cardiac autonomic function [20]. Similarly, a community consisting of older men showed that a higher intake of green leafy vegetables, rich in vitamin C and carotenoids, had a beneficial effect on autonomic cardiac function [21]. In addition, a randomized cross-over study of healthy subjects showed the protective effect of passion fruit juice supplementation on cardiac autonomic responses [22]. Long-term vegetarian diets are also suggested to be positively associated with a higher high-frequency power of heart rate variability [23]. However, there is no previous studies addressing the role of vegetable and fruit diversity consumption on ANS activity with children. Moreover, previous results suggest that diet might change the ANS response, which is important to maintain physiologic functions involving cardiovascular, respiratory, and gastrointestinal systems [24], however, the underlying mechanisms that might explain the association between diet and disease risk in children are not fully understood.

In this context, a greater consumption of fruit and vegetable varieties, rich in several bio-compounds, might be implicated in the ANS regulation. Therefore, we aimed to evaluate the association between the diversity of vegetable and fruit consumption with the ANS activity in school-aged children.

## 2. Methods

### 2.1. Study Design and Participants

The current secondary analysis included 513 children (49.9% girls) aged 7 to 12 years. These children participated in a previous cross-sectional study that included 858 participants from 71 classrooms within 20 public schools located in city of Porto, Portugal, from January 2014 to January 2015, as detailed elsewhere [25].

The study was approved by the Ethical Committee (ARIA 248-13), procedures were in accordance with Helsinki Declaration, and written consent was obtained from the children’s legal guardians.

### 2.2. Participants Assessment

#### 2.2.1. Pupillometry

Pupillometry was carried out with a portable infrared PLR-200pupillometer (NeurOptics PLR-200™ Pupillometer, NeurOptics Inc., Irvine, CA, USA), as previously described [25]. We recorded a pupillary light response for each eye, and no differences between them were found. Seven pupillometry measures were assessed. The initial diameter of the pupil (baseline pupil diameter), the pupil at constriction peak (final pupil diameter) (millimeters (mm)), relative constriction amplitude (%), and average constriction and dilation velocities (ACV and ADV, respectively, mm/s) were associated with the parasympathetic nervous activity [24]. The maximum constriction velocity (MCV, mm/s) and total time taken by the pupil to recover to 75% of the initial resting pupil size after reaching peak of constriction (T75, seconds) were associated with sympathetic nervous activity [26]. The pupil was controlled by the sympathetic and parasympathetic branches of the ANS [27], and pupillometry was applied to assess the differences in pupillary light response between healthy individuals and patients with several pathologies, including autonomic neuropathy [28], diabetes [29], and Parkinson’s disease [30].

#### 2.2.2. Vegetable and Fruit Diversity

According to standard procedures, the child’s dietary intake in the prior 24 h was discovered with a 24-h recall questionnaire for the children, which allowed us to obtain detailed data about the consumption of foods and beverages. The total intake of vegetable and fruit (in grams) consumed in one day was assessed through the Food Processor^®^ software (ESHA Research, Salem, OR, USA), which includes databases of Portuguese nutritional food-composition.

The Food and Agriculture Organization (FAO) of the United Nations proposed a dichotomous indicator to assess diet diversity that accounts for the consumption of at least 5 out of 10 food-groups included [31]. To calculate the vegetable and fruit diversity score, we considered the “Pulses (beans, peas and lentils)”, “Dark green leafy vegetables”, “Other vitamin A-rich fruits and vegetables”, “Other vegetables” and “Other fruits”. Two different scores were built based on the consumption of at least 15 g of fruits and vegetables [31]. As previously described [12] one of the scores—the vegetable diversity—was calculated based on the sum of different pulses, dark green leafy vegetables, other pro-vitamin-A-rich vegetables, and other vegetables (those not included in the previous group) consumed in one day. The other score—the fruit diversity score—was calculated based on the sum of different pro-vitamin A-rich fruits and other fruits. Mixed dishes, such as soup or fruit salad, were considered as one different item.

#### 2.2.3. Anthropometry

Body mass index (BMI) was calculated from the weight, which was measured by a digital scale (Tanita™ BC-418 Segmental Body Analyzer) and recorded in kilograms (kg), and height, which was measured by a portable stadiometer, recorded in centimeters (cm), and presented as kg per square meters (kg/m^2^). The age- and sex-specific percentiles from the US Centers for Disease Control and Prevention (CDC) was used to categorize participants [32]. This definition was considered based on prior assessment of the degree of agreement between different classifications of BMI (US CDC, World Health Organization, International Obesity Task Force, and Percentage of body fat), where the US CDC present the highest degree of agreement with all the other classifications (data not shown).

#### 2.2.4. Statistical Analyses

The characteristics of the participants are presented for the whole sample by sex as percentages for categorical, and as median (25th–75th percentile) for continuous variables, except for age which is expressed as mean ± standard deviations (SD). Differences between groups were assessed through the Mann–Whitney test (Student’s *t*-test for independent variables for age) and Chi-squared test for continuous and categorical variables, respectively.

Linear regression models were used to assess the association between vegetable and fruit diversity scores with pupillometry measures, and the magnitude of the association was measured using linear coefficients (β) and its respective 95% confidence interval (CI). Age, sex, body mass categories, and daily vegetable intake or daily fruit intake were considered as confounders. The effect modification of the child’s sex on the association between vegetable and fruit diversity scores with pupillometry was examined by a product term added into fully adjusted models, but the associations were not significant. Variance inflation factor was used to measure the degree of multicollinearity between independent variables, and values below 10 were observed.

A 0.05 level of significance and 95% CI was found. The analyses were performed through the SPSS statistical package software v26.0 (IBM, Armonk, NY, USA).

## 3. Results

The main characteristics of the participants are presented in Table 1. The median vegetable and fruit diversity score was 2.5 (2.0; 3.0) and 1.0 (1.0; 2.0), respectively. The median intake of vegetables and fruits was 403.0 g (25th–75th percentiles: 290.0; 625.0) and 234.5 g (150.0; 342.0), respectively. No significant differences between boys and girls were found.

Increasing the daily consumption of vegetables was positively associated with ADV (crude model, β = 0.45, 95%CI: 0.09; 0.81, Table 2). After adjustment for sex, age, body mass categories, and daily vegetable intake, the association remained significant with ADV (β = 0.03, 95%CI: 0.002; 0.07, Table 2). No significant associations were found between fruit diversity consumption and pupillometry measures (Table 2).

## 4. Discussion

We found that greater vegetable diversity consumption was positively associated with the average dilation velocity, a pupillometry measure associated with the SNS activity. No evidence of association was found between the ANS and fruit diversity consumption. Nonetheless, the composition of vegetables and fruits may differ significantly for several bioactive components and nutrients, especially in sugar content—fructose. Therefore, it is possible that vegetable diversity influences the ANS response in a different way when compared to fruit diversity consumption. In line with our findings, despite the methodological differences on outcome assessment, current evidence suggests that polyphenol-rich foods might be associated with the SNS over-activity [6]. A study conducted in male twins showed that a higher Mediterranean diet score, which is characterized by a higher vegetable and fruit intake was also positively associated with both time and frequency domain measures of HRV, namely, total power, ultra-low frequency, very-low frequency, and low frequency power, suggesting a modulation of the parasympathetic and sympathetic nervous activity [20]. In addition, results from a Canadian cohort showed that mothers with poorer diet quality during pregnancy, which was assessed through the Healthy Eating Index (2010), had infants with lower HRV, including the root mean square of successive differences (B = 0.07; 95% CI: 0.01, 0.13) and SD of N-N intervals (B = 0.18; 95% CI: 0.02, 0.35) [33].

Studies exploring responses to food based on visual, odor, and taste cues and its effect on ANS have also been explored. de Wijk et al. found that “liked foods” were positively associated with skin conductance response (SCR) and finger temperature (FT), both proxy measures of SNS activity [34]. Apparently, the combination of the increase of both SCR and FT is associated with emotions of anticipated pleasure, suggesting that the responses from the ANS objectively reflect the individual’s emotional reactivity. Accordingly, Anderson et al., proposed that an increased activity of the SNS may be associated with a pleasant meal, which will be different for each person’s experience, since it is dependent of internal sensations based on previous experiences and knowledge [35]. On the other hand, a study characterizing primary tastes based on ANS responses found that the consumption of unpleasant-connoted tastes, including salty tastes, were strong inducers of electrodermal, thermovascular, and cardiac responses [36].

Diet quality might be reflected on the diversity of food groups consumed and the recommendation to have a more diverse diet is based on the premise that consuming a variety of foods may increase nutrient adequacy and reduce the risk of non-communicable diseases. The consumption of vegetables and fruits has been widely promoted considering its high content in several vitamins, minerals, and phytochemicals, such as anti-oxidants [37]. The SNS is critically influenced at the central and peripheral level, by important factors, such as reactive oxygen species, but the mechanisms that explain how oxidative stress influences its sympatho-excitatory effects are not well-established [6]. Nevertheless, our findings show that consuming an increased amount of different vegetables per day might impact the SNS activity, which might be explained by the potential additive and synergistic effect resulting from the combination of different dietary components with anti-inflammatory and anti-oxidant properties. In turn, these dietary components, including dietary fiber, micronutrients, and bioactive compounds that participate on important activities of the gut microbiota, low-grade inflammation, and of preventing cells from oxidative stress [38], might exercise their effect on ANS activity. As such, a higher vegetable diversity consumption might influence the ANS response, a possible mechanism that might explain the association between diet and non-communicable diseases.

This study has some limitations. Firstly, the cross-sectional design does not allow establishing causal relationships. Secondly, diversity scores were evaluated using a single 24-h recall questionnaire. The assessment of children’s diet is challenging, particularly without the parent’s presence. However, the 24-h recall questionnaire seems to be valuable to capture important information about individual food and drink intake [39]. Although the dietary data might be affected by a recall bias, where children might find difficulties in self-reporting their diet considering their incomplete knowledge about what they eat, children can more easily report the foods and beverages consumed in the prior 24 h. Regarding fruit and vegetable intake, Haraldsdóttir et al. showed that in Portuguese children, the 24-h recall part from a questionnaire that also included a food frequency part, provided valid estimates for the mean intake of vegetables, while it overestimated the intake of fruits [40]. Nonetheless, the assessment of diversity of fruit and vegetable consumption has been performed by a single 24-h recall [11]. Thirdly, as we previously suggested [12], the median of different fruits consumed in one day within our study sample is one, which does not reflect diversity. As proposed by Almeida-de-Souza et al., a fruit variety more homogeneous, which reflects a lower standard deviation (SD), is less discriminant on showing low-grade inflammation [13]. Similarly, our results showed that vegetable diversity was less homogeneous when compared to fruit diversity (mean = 2.57 ± 1.26 vs. 1.32 ± 0.95, data not shown), suggesting that vegetable diversity consumption might be more discriminant on showing ANS response modulation. Fourthly, the study considered a single pupillometry measure. However, the pupillary responses are involuntary [41], and previous studies found that pupil responses were stable after repeated measurements during a single test session [42]. In addition, despite the fact that previous research showed that this test could be performed to identify an early autonomic dysfunction; there is no standardization and consensus of testing protocols [42]. Nonetheless, a study conducted in children found that quantitative pupillometry was able to provide information in more detail, compared to the traditional characteristics evaluated during a physical examination [43]. Pupillometry also proved to be more suitable for detecting subtle changes in the pupil, suggesting that this might be more relevant to provide sensitive information [43,44,45]. Moreover, it was recently found that pupil size is correlated with measures of HR, considerably controlled by the PSNS and galvanic skin response, an independent index of sympathetic activity [46]. However, a study suggested that there is a maturation of the pupillary response according to the age and sex among healthy children [47]. Lastly, we take into consideration different confounders, but we cannot exclude the potential impact from other unmeasured covariates on our findings.

Our study also has important strengths. To our knowledge, this study is the first observational study performing the evaluation of the association between vegetable and fruit diversity consumption with ANS assessed through pupillometry in school-aged children. It is also the first work suggesting a plausible mechanism through which diet might modulate the ANS in school-aged children. Moreover, we used pupillometry to assess the ANS, which has been used as a valid low-cost and non-invasive tool to assess sympathetic and parasympathetic activity [48,49], surpassing the limitations regarding technology and signals of frequently used tools [50]. Furthermore, this study included a large number of participants, whose data were collected in detail by the same research team.

## 5. Conclusions

Our findings show that vegetable diversity intake modulated the ANS response in children, a possible early link between diet and health in school-aged children. Additional studies are needed to further understand the underlying mechanisms through which diet might modulate the ASN.

## Figures and Tables

**Table 1:** Characteristics of the participants.

	Girls, *n* = 256	Boys, *n* = 257	Total, *n* = 513	*p*-Value
Age (years), ±SD	8.80 ± 0.80	8.81 ± 0.81	8.80 ± 0.80	0.979
BMI categories ^1^, *n* (%)				
Underweight	14 (5.50)	10 (3.90)	24 (4.70)	0.700
Normal weight	179 (69.90)	181 (70.60)	360 (70.20)	
Overweight	37 (14.50)	34 (13.20)	71 (13.80)	
Obese	26 (10.20)	34 (13.20)	58 (11.30)	
Daily vegetable intake (g)	404.5 (290.0; 595.0)	420.0 (279.0; 685.9)	403.0 (290.0; 625.0)	0.385
Daily fruit intake (g)	232 (158.0; 328.0)	250.0 (138.0; 348.0)	234.5 (150.0; 342.0)	0.839
Vegetable diversity score	2.5 (2.0; 3.0)	2.5 (2.0; 3.0)	2.5 (2.0; 3.0)	0.511
Fruit diversity score	1.0 (1.0; 2.0)	1.0 (1.0; 2.0)	1.0 (1.0; 2.0)	0.763
Pupillometry				
Baseline pupil diameter (mm)	5.40 (4.70; 4.90)	5.40 (4.90; 5.90)	5.40 (4.80; 5.90)	0.648
Final pupil diameter (mm)	3.45 (3.0; 3.90)	3.40 (3.0; 3.80)	3.40 (3.0; 3.90)	0.729
ACV (mm/s)	4.04 (3.57; 4.45)	4.05 (3.63; 4.45)	35.0 (33.0; 38.0)	0.354
ADV (mm/s)	1.14 (0.98; 1.32)	1.17 (0.99; 1.33)	1.15 (0.99; 1.32)	0.545
Constriction amplitude (%)	35.0 (32.0; 38.0)	36.0 (33.0; 39.0)	35.0 (33.0; 38.0)	0.055
MCV (mm/s)	5.32 (4.68; 5.85)	5.41 (4.85; 5.94)	5.37 (4.76; 5.88)	0.112
T75 (s)	1.67 (1.17; 2.07)	1.67 (1.23; 2.13)	1.67 (1.20; 2.13)	0.652

^1^ according to US Centers for Disease Control; Data are expressed as medians (25th–75th percentile) unless otherwise stated. Baseline pupil diameter: initial diameter of the pupil before. Final pupil diameter: diameter of the pupil at constriction peak. ACV: average constriction velocity. ADV: average dilation velocity; relative constriction amplitude. MCV: maximum constriction velocity. T75: total time taken by the pupil to recover to 75% of the initial resting pupil size after reaching peak of constriction.

**Table 2:** Association between vegetable and fruit diversity consumption and pupillometry measures (β-coefficients and 95% CI).

	Vegetable Diversity		Fruit Diversity	
		R^2^		R^2^
Baseline pupil diameter				
Crude Model	0.12 (−0.01; 0.25)	0.006	0.02 (−0.06; 0.09)	0.0004
Adjusted Model	0.007 (−0.07; 0.08)	0.004	0.03 (−0.09; 0.15)	0.014
Final pupil diameter				
Crude Model	0.19 (0.01; 0.37)	0.009	0.02 (−0.04; 0.07)	0.001
Adjusted Model	0.02 (−0.04; 0.07)	0.009	0.03 (−0.06; 0.11)	0.015
ACV				
Crude Model	−0.02 (−0.19; 0.15)	0.001	0.03 (−0.03; 0.09)	0.002
Adjusted Model	−0.04 (−0.10; 0.02)	0.007	0.03 (−0.07; 0.12)	0.004
ADV				
Crude Model	0.45 (0.09; 0.81)	0.01	−0.01 (−0.04; 0.02)	0.001
Adjusted Model	0.03 (0.002; 0.07)	0.047	0.01 (−0.04; 0.05)	0.046
Constriction amplitude				
Crude Model	−0.01 (−0.04; 0.01)	0.003	−0.10 (−0.55; 0.34)	0.0004
Adjusted Model	−0.18 (−0.62; 0.27)	0.018	−0.09 (−0.72; 0.55)	0.011
MCV				
Crude Model	−0.02 (−0.15; 0.10)	0.0003	0.04 (−0.04; 0.12)	0.002
Adjusted Model	−0.02 (−0.10; 0.06)	0.009	0.05 (−0.08; 0.17	0.009
T75				
Crude Model	0.04 (−0.13; 0.21)	0.001	0.01 (−0.06; 0.08)	0.0002
Adjusted Model	−0.001 (−0.07; 0.07)	0.005	0.04 (−0.07; 0.15)	0.017

Baseline pupil diameter: initial diameter of the pupil before. Final pupil diameter: diameter of the pupil at constriction peak. ACV: average constriction velocity. ADV: average dilation velocity; relative constriction amplitude. MCV: maximum constriction velocity. T75: total time taken by the pupil to recover to 75% of the initial resting pupil size after reaching peak of constriction. Adjusted model: adjusted for sex, age, body mass categories, and daily vegetable intake or daily fruit intake.

## Data Availability

The data presented in this study are available on reasonable request from the corresponding author. The data are not publicly available due to ethical requirements.
